# Putting focus on transcranial direct current stimulation in language production studies

**DOI:** 10.1371/journal.pone.0202730

**Published:** 2018-08-23

**Authors:** Jana Klaus, Dennis J. L. G. Schutter

**Affiliations:** Donders Institute for Brain, Cognition and Behaviour, Radboud University, Nijmegen, The Netherlands; University Medical Center Goettingen, GERMANY

## Abstract

Previous language production studies targeting the inferior frontal and superior temporal gyrus using anodal tDCS have provided mixed results. Part of this heterogeneity may be explained by limited target region focality of conventionally used electrode montages. We examined the focality of conventionally and alternative electrode montages. Electrical field distributions of anodal tDCS targeting IFG and pSTG were simulated in conventional setups (anodal electrode over left IFG/pSTG, reference electrode over right supraorbital region) and an alternative electrode montage in four different brains. Conventional montages showed maximum field strengths outside of the target regions. Results from alternative electrode montages showed that focality of tDCS could be improved by adjustments in electrode placement. Heterogeneity of findings of language production studies deploying conventional montages may in part be explained by diffuse electrical field distributions. Alternative montages may improve focality and provide more unequivocal results.

## Introduction

In studies on the functional neuroanatomy of language production transcranial direct current stimulation (tDCS) is routinely administered over the left inferior frontal gyrus (IFG) or posterior superior temporal gyrus (pSTG) in healthy volunteers. Results of these non-invasive brain stimulation studies have been subject to a significant degree of variability. For example, a number of studies reported a beneficial effect of anodal tDCS as evidenced by higher verbal fluency scores or shorter response times in picture naming tasks [1–6], while others did not find such an improvement [7–11]. Furthermore, meta-analyses on the ability of tDCS to modulate language performance in healthy volunteers have reported small or no effects [12–15]. Thus, even though tDCS may be effective in establishing effects on language processes, the heterogeneous results illustrate the difficulties associated with tDCS in anticipating both the direction and the magnitude of its behavioural effects [16,17].

Issues concerning the spatial resolution of the induced electrical field are considered to be among the most important contributors to the diversity of tDCS-related effects. The vast majority of studies routinely placed one electrode over either the IFG or pSTG and the return electrode over the right supraorbital region (rSO, e.g. [1,8,11,18–20]). Even though these montages have demonstrated the potential to be effective in manipulating processes underlying language production, computational simulation studies indicate that the intracranial electrical field distribution of tDCS is diffuse and the peak field strength amplitude is not located directly underneath the electrode [21]. For instance, it has been shown that the maximum field strength between two electrodes is obtained if the target electrode is approximately placed between 20 and 40 mm away from the target region [21,22]. By this logic, placing the active electrode directly over the target site may induce a maximum field strength in regions adjacent to the targeted area. As a result, attributing changes in language production that are assumed to be caused by manipulating the regions of interest directly under the electrode can become more difficult. In spite of the well documented fact that electrode montage is an important aspect of tDCS experiments [22–24], the extent to which suboptimal electrode montages may at least partially account for the heterogeneity of results in language production studies has not been examined yet. The goal of the present study was to (1) provide an estimate of the electrical field distributions of the two most commonly used electrode montages targeting the IFG and pSTG in language production tDCS studies, and (2) to present alternative ways to optimize the use of tDCS in language production studies.

## Material and methods

### Participants

We used the T1-weighted resting-state structural magnetic resonance images from four participants provided in the publicly available dataset of the Sleepy Brain project [25]. We selected a male and female student from a young sample (between 20 and 30 years; participants 9001 and 9018 in the original dataset) and a male and female participant from an old sample (between 65 and 75 years; participants 9002 and 9004) to cover potential variability caused by age and sex differences.

### Procedure

All computations were run with the SimNIBS software (version 2.0 [26]). We first created individual tetrahedral head models using the mri2mesh algorithm implemented in the SimNIBS pipeline (see [27] for a more specific description of the procedure). Then we simulated the electrical field distribution of anodal 1.5 mA tDCS with 5 × 7 cm electrodes (current density: 0.043 mA/cm^2^) in four different scenarios on the four different brains. For all simulations, we chose an electrode-sponge setup, with a 1 mm thick electrode covered by a 2 mm thick sponge.

### Computations

First we tested whether the conventionally used montage of placing the active electrode over the target region and the reference electrode over the contralateral supraorbital region provides the desired focality across the left IFG and pSTG, respectively. To address the second aim of our study, we used the computational findings reported by Rampersad et al. [21] to adjust the electrode positions in the following ways (1) moving the centre of the active electrode approximately 3 cm anterior to the target region; (2) placing the reference electrode in closer proximity to the active electrode; (3) turning the electrode so that the short edges of both electrodes approximately face each other. Electrode size was kept constant in order to directly compare the influence of electrode placement on the focality of tDCS.

## Results

Table 1 reports the individual minimum and maximum electrical field strengths (in V/m) broken down by stimulation site (IFG vs. pSTG) and montage (conventional vs. alternative). Note, however, that these values refer to field strengths observed anywhere in the brain and do not speak to the question which electrical fields are elicited in the target regions. Nevertheless, it does illustrate both inter- and intraindividual variability in the magnitude of the generated electrical field, as has also been reported in a large sample by Laakso et al. [22].

**Table 1:** Minimum and maximum electrical field strengths (in V/m) for all simulations.

	IFG				pSTG			
	Standard montage		Alternative montage		Standard montage		Alternative montage	
	*min*	*max*	*min*	*max*	*min*	*max*	*min*	*max*
**Young male**	0.011	0.673	0.010	0.787	0.018	0.809	0.011	0.569
**Young female**	0.012	0.498	0.010	0.691	0.020	0.599	0.014	0.475
**Old male**	0.010	0.998	0.014	0.703	0.021	1.109	0.016	0.517
**Old female**	0.009	0.725	0.011	1.160	0.017	0.925	0.015	0.532

### IFG montage

**Fig 1:**
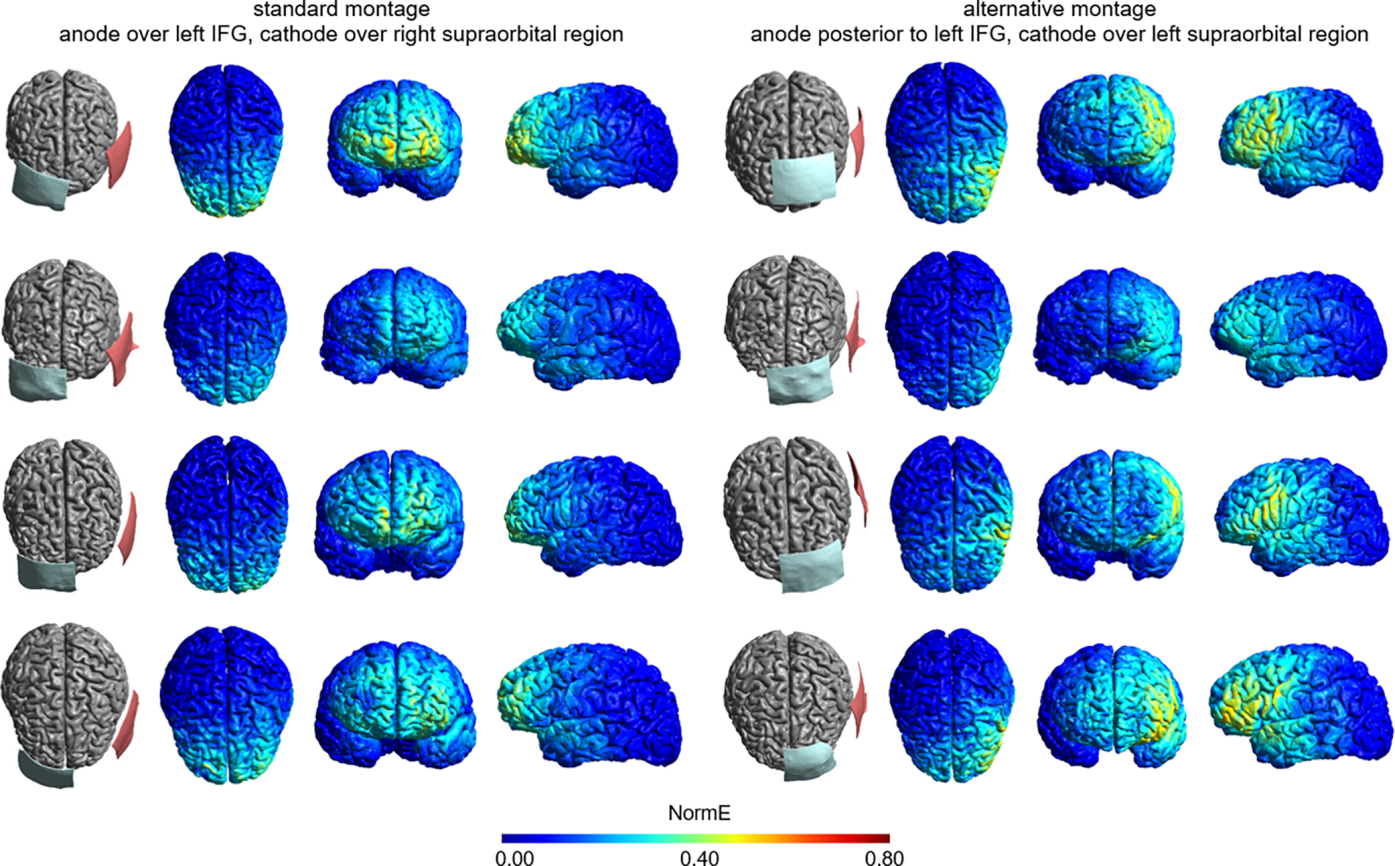
Electrode montages and electrical field intensities for tDCS targeting the left inferior frontal gyrus for the four participants (from top to bottom: Young male, young female, old male, old female). The left part displays the simulation results for the conventional montage in which the anodal electrode is placed over the left IFG and the cathodal electrode is placed over the right supraorbital region. The right part displays the simulation results for the alternative montage in which the anodal electrode is placed posterior to the left IFG and the cathodal electrode is placed over the left supraorbital region. All electrical fields are scaled between 0 and 0.8 V/m, with brighter colours indicating higher electrical field strengths.

Fig 1 displays the electrode montages and simulation results for the montages targeting the left IFG in the four brains. Results indicate that the conventionally used montage (left panel of Fig 1) is not optimal as peaks in field strength were found in bilateral middle and superior frontal regions. Furthermore, the field distributions spread with decreasing intensity to left central and anterior temporal regions. Thus, while the standard montage did affect the target region, substantially higher electrical fields were observed anterior to the region of interest, and in both hemispheres.

The alternative electrode montage (right panel of Fig 1) showed a notable shift of the electrical field. While the magnitude and spread of the induced electrical field differed between individuals, the simulations uniformly displayed a convergence to the target area. That is, the effect on the right hemisphere was reduced, and the peak intensities were located around the IFG and the central sulcus, with additional, decreased field intensities in middle frontal and anterior temporal regions.

### pSTG montage

**Fig 2:**
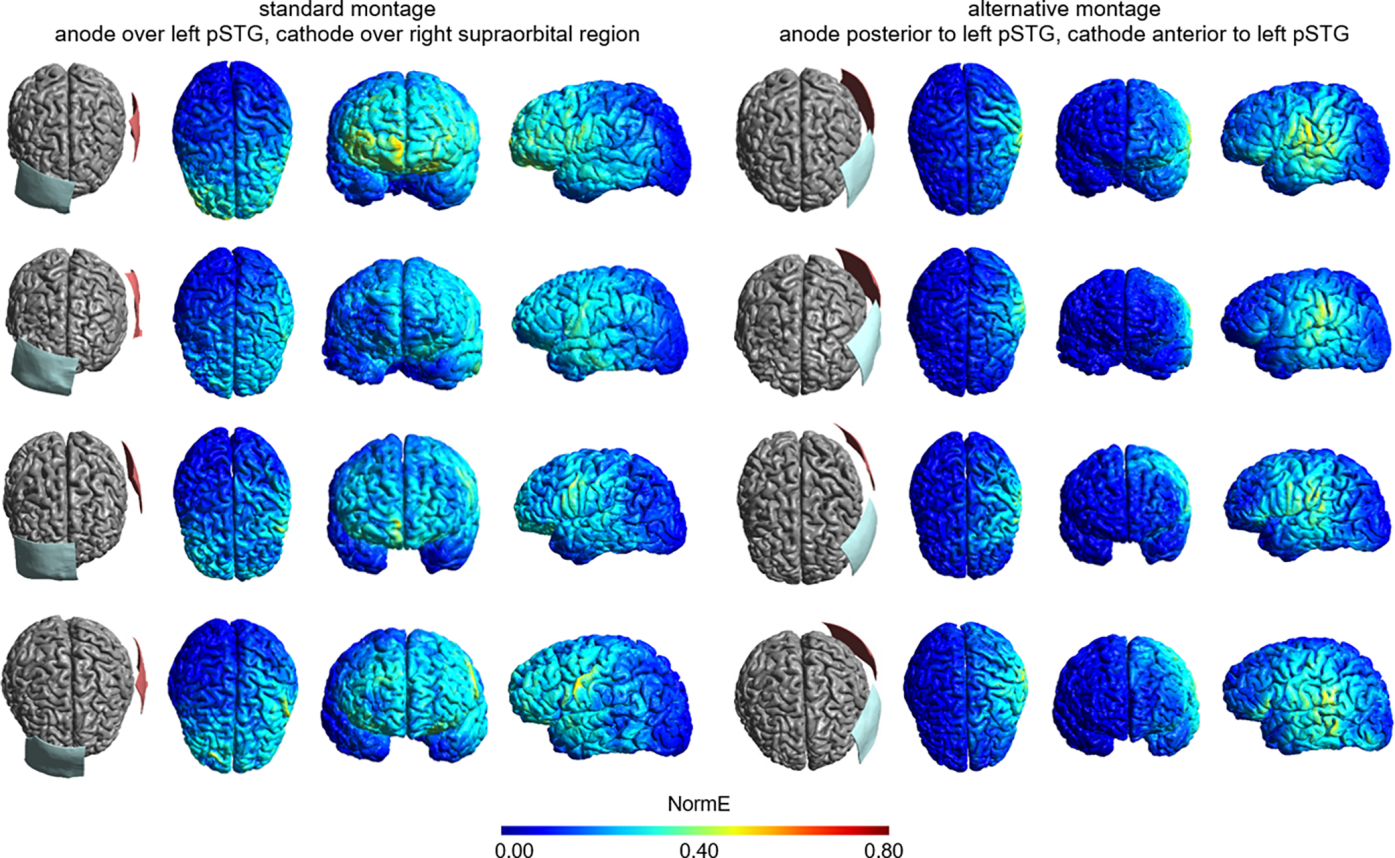
Electrode montages and electrical field intensities for tDCS targeting the left posterior superior temporal gyrus for the four participants (from top to bottom: Young male, young female, old male, old female). The left part displays the simulation results for the conventional montage in which the anodal electrode is placed over the left pSTG and the cathodal electrode is placed over the right supraorbital region. The right part displays the simulation results for the alternative montage in which the anodal electrode is placed posterior to the left pSTG and the cathodal electrode is placed anterior to the left pSTG. All electrical fields are scaled between 0 and 0.8 V/m, with brighter colours indicating higher electrical field strengths.

Fig 2 shows the results from simulations targeting the pSTG. The conventional montage (left part of Fig 2) resulted in a wide electrical field distribution in the left hemisphere. Field strength peaks were centred on the medial part of the postcentral gyrus (i.e., anterior to the target region). Additionally, the induced electrical field covered significant parts of the left hemisphere and anterior parts of the contralateral hemisphere, arguably due to the large distance between the electrodes. As for IFG stimulation, the alternative montage (right panel of Fig 2) shifted the field intensity peaks towards the target region, with the highest field strength found in a rather large region including the pSTG and the inferior parietal lobule. Right-hemispheric effects were eliminated entirely because for pSTG stimulation, the alternative montage exclusively placed the electrodes across the left hemisphere, thus preventing the generation of electric fields in the right hemisphere.

## Discussion

Our simulation results showed that the electrical field distribution of tDCS montages most commonly used in language production studies can be improved. Placement of the active electrode over the target region and the reference electrode over the contralateral supraorbital region yields the highest field strengths anterior to the target region as well as additional frontal effects in the right hemisphere. These wide electrical field distributions may cause collateral activation of surrounding tissue and contribute to the heterogeneous findings reported in previous studies. While there is no immediate reason to assume that the target regions were not exposed to the exogenous electrical field at all in previous studies, conventionally applied montages may have been suboptimal in reaching the desired spatial resolution of the electrical field. Consequently, small effects of tDCS on language production may have been caused, at least in part, by affecting the target region to varying degrees.

Here, we provide an alternative montage that based on computer simulations produced more focal peaks in electrical field strength. Altering the montage by placing the electrodes anterior and posterior to the target region improved focality in all of our four brains. Using such a montage may thus target the desired region more directly and limit electrical field exposure of surrounding regions. As a result, future studies examining differences between real and sham tDCS conditions may find more reliable and unequivocal effects in language production tasks. The magnitude of the induced electrical field appears to be well in the range required to elicit cellular effects [28]. Nevertheless, it remains to be tested whether the improved montages are indeed effective in obtaining less unequivocal effects of tDCS in language production performance. Additionally, as can be seen from the simulations, the cortical area affected by the stimulation typically covers a large region between the electrodes. It remains to be tested whether additional modifications to the montages (e.g. by using smaller electrodes or a high-definition tDCS setup) further reduce induced field strengths in regions peripheral to the target region.

Aside from the basic neuroscientific questions on language production, tDCS is currently being explored as a possible therapeutic intervention to treat aphasia [29–31]. Based on our findings, it is reasonable to assume that aphasic patients treated with tDCS may benefit from adopting simulation-based electrode montages to maximize focal electrical field strengths (see also [32] for a simulation-based approach in a lesioned brain). However, in these clinical cases, the cortical factors causing the language disorder may play a crucial role in determining the efficacy of tDCS treatments. That is to say, patients with post-stroke aphasia, in which varyingly large areas of the left hemisphere are chronically lesioned, may respond differently to (optimised) tDCS montages than patients whose language deficit is the result of a neurogenerative disease (e.g. in primary progressive aphasia). As such, it is important to further investigate whether the efficacy of different tDCS montages can be extrapolated to different disorders, or whether some might indeed continue to require individual modelling to allow for the best possible outcome.

It should be noted that the simulations were performed on a small number of brains, which limits the generalisability of our results. Evidently, the efficacy of tDCS depends on many factors that include individual differences in gyral folding, thickness of the cerebrospinal fluid and skull bone, and physiological susceptibility to weak electrical currents [26,33]. These sources of variability are also visible in the current simulations, in that neither the conventional nor the alternative montages cause a uniform current spread across individuals, but instead vary both in terms of the spatial resolution and the magnitude of the electrical field. Nevertheless, we have demonstrated that despite these individual differences in electrical field distributions, the alternative montages were consistently more successful in targeting the desired regions. Thus, irrespective of anatomical and cellular differences between participants, our simulations provide evidence that the standard montage is suboptimal and alternative montages may be able to at least approximate a more targeted application of tDCS. Finally, research labs and clinics that do not have direct access to neuroimaging facilities to individualize tDCS montages, simulations provide a pragmatic solution that outweighs the alternative of relying on conventionally used montages.

Importantly, optimizing the electric field distribution is not the only determinant of the effective modulation of cortical activity. For instance, Pisoni and colleagues [34] elegantly illustrated the neurophysiological effects of applying anodal tDCS over the left IFG while participants performed a verbal fluency task. In a combined TMS-EEG setup, they found that compared to sham tDCS, anodal tDCS resulted in a functionally specific increase of cortical excitability in BA 6 and BA 44/45. Moreover, this increase was positively correlated with the performance increase in the verbal fluency task, implying that participants in whom tDCS caused greater cortical excitability in BAs 6 and 44/45 also showed the largest performance improvement. Overall, these results suggest that effective (i.e. performance-enhancing) tDCS is not limited to spatial specificity, but also to physiological susceptibility of brain tissue. An interesting endeavour would be to investigate whether these results can be replicated and/or amplified with the alternative montage proposed in the current study, ultimately leading to a more detailed picture of which parameters to modulate to get the most robust neuromodulatory effects.

Furthermore, implicit expectations by the participants regarding the effect of the stimulation may add to previously reported variability. Using a reinforcement learning paradigm, it has recently been shown that the mere prospect of tDCS positively affecting behavioural performance indeed causes performance improvements, even if sham tDCS is applied [35]. This finding raises another important issue in tDCS research, namely that assumptions about the efficacy of the treatment—potentially even when conveyed unconsciously by the experimenter—can alter the behavioural outcome.

In conclusion, our results show that the type of montage may contribute to the robustness of findings, improve their interpretations, and advance the application of tDCS on language production performance in basic neuroscientific and clinical settings.

## References

[1] Cattaneo Z, Pisoni A, Papagno C. Transcranial direct current stimulation over Broca’s region improves phonemic and semantic fluency in healthy individuals. Neuroscience. 2011;183: 64–70. 10.1016/j.neuroscience.2011.03.058 21477637

[2] Fertonani A, Rosini S, Cotelli M, Rossini PM, Miniussi C. Naming facilitation induced by transcranial direct current stimulation. Behav Brain Res. 2010;208: 311–318. 10.1016/j.bbr.2009.10.030 19883697

[3] Holland R, Leff AP, Josephs O, Galea JM, Desikan M, Price CJ, et al Speech facilitation by left inferior frontal cortex stimulation. Curr Biol. 2011;21: 1403–1407. 10.1016/j.cub.2011.07.021 21820308 PMC3315006

[4] Meinzer M, Antonenko D, Lindenberg R, Hetzer S, Ulm L, Avirame K, et al Electrical brain stimulation improves cognitive performance by modulating functional connectivity and task-specific activation. J Neurosci. 2012;32: 1859–1866. 10.1523/JNEUROSCI.4812-11.2012 22302824 PMC6703352

[5] Sparing R, Dafotakis M, Meister IG, Thirugnanasambandam N, Fink GR. Enhancing language performance with non-invasive brain stimulation—A transcranial direct current stimulation study in healthy humans. Neuropsychologia. 2008;46: 261–268. 10.1016/j.neuropsychologia.2007.07.009 17804023

[6] Vannorsdall TD, Schretlen DJ, Andrejczuk M, Ledoux K, Bosley L V, Weaver JR, et al Altering automatic verbal processes with transcranial direct current stimulation. Front Psychiatry. Frontiers Media SA; 2012;3: 73 10.3389/fpsyt.2012.00073 22888321 PMC3412390

[7] Cerruti C, Schlaug G. Anodal transcranial direct current stimulation of the prefrontal cortex enhances complex verbal associative thought. J Cogn Neurosci. NIH Public Access; 2009;21: 1980–7. 10.1162/jocn.2008.21143 18855556 PMC3005595

[8] Ehlis A-C, Haeussinger FB, Gastel A, Fallgatter AJ, Plewnia C. Task-dependent and polarity-specific effects of prefrontal transcranial direct current stimulation on cortical activation during word fluency. Neuroimage. 2016;140: 134–140. 10.1016/j.neuroimage.2015.12.047 26748077

[9] Henseler I, Mädebach A, Kotz SA, Jescheniak JD. Modulating brain mechanisms resolving lexico-semantic interference during word production: A transcranial direct current stimulation study. J Cogn Neurosci. 2014;26: 1403–1417. 10.1162/jocn_a_00572 24405107

[10] Vannorsdall TD, van Steenburgh JJ, Schretlen DJ, Jayatillake R, Skolasky RL, Gordon B. Reproducibility of tDCS results in a randomized trial. Cogn Behav Neurol. 2016;29: 11–17. 10.1097/WNN.0000000000000086 27008245

[11] Westwood SJ, Olson A, Miall RC, Nappo R, Romani C. Limits to tDCS effects in language: Failures to modulate word production in healthy participants with frontal or temporal tDCS. Cortex. 2017;86: 64–82. 10.1016/j.cortex.2016.10.016 27912107 PMC5264390

[12] Horvath JC, Forte JD, Carter O. Quantitative review finds no evidence of cognitive effects in healthy populations from single-session transcranial direct current stimulation (tDCS). Brain Stimul. 2015;8: 535–550. 10.1016/j.brs.2015.01.400 25701175

[13] Klaus J, Schutter DJLG. Non-invasive brain stimulation to investigate language production in healthy speakers: A meta-analysis. Brain Cogn. 2018;123: 10–22. 10.1016/j.bandc.2018.02.007 29499493

[14] Price AR, McAdams H, Grossman M, Hamilton RH. A meta-analysis of transcranial direct current stimulation studies examining the reliability of effects on language measures. Brain Stimul. 2015;8: 1093–1100. 10.1016/j.brs.2015.06.013 26210573 PMC4833093

[15] Westwood SJ, Romani C. Transcranial direct current stimulation (tDCS) modulation of picture naming and word reading: A meta-analysis of single session tDCS applied to healthy participants. Neuropsychologia. 2017; 10.1016/j.neuropsychologia.2017.07.031 28757003

[16] Jacobson L, Koslowsky M, Lavidor M. tDCS polarity effects in motor and cognitive domains: a meta-analytical review. Exp Brain Res. Springer-Verlag; 2012;216: 1–10. 10.1007/s00221-011-2891-9 21989847

[17] Oldrati V, Schutter DJLG. Targeting the human cerebellum with transcranial direct current stimulation to modulate behavior: A meta-analysis The Cerebellum. Springer US; 2017; 1–9. 10.1007/s12311-015-0758-5 PMC5849643 28786014

[18] Fiori V, Cipollari S, Caltagirone C, Marangolo P. “If two witches would watch two watches, which witch would watch which watch?” tDCS over the left frontal region modulates tongue twister repetition in healthy subjects. Neuroscience. 2014;256: 195–200. 10.1016/j.neuroscience.2013.10.048 24184977

[19] Pisoni A, Cerciello M, Cattaneo Z, Papagno C. Phonological facilitation in picture naming: When and where? A tDCS study. Neuroscience. 2017;352: 106–121. 10.1016/j.neuroscience.2017.03.043 28385634

[20] Pisoni A, Papagno C, Cattaneo Z. Neural correlates of the semantic interference effect: New evidence from transcranial direct current stimulation. Neuroscience. 2012;223: 56–67. 10.1016/j.neuroscience.2012.07.046 22863670

[21] Rampersad SM, Janssen AM, Lucka F, Aydin U, Lanfer B, Lew S, et al Simulating transcranial direct current stimulation with a detailed anisotropic human head model. IEEE Trans Neural Syst Rehabil Eng. 2014;22: 441–452. 10.1109/TNSRE.2014.2308997 24760939

[22] Laakso I, Tanaka S, Mikkonen M, Koyama S, Sadato N, Hirata A. Electric fields of motor and frontal tDCS in a standard brain space: A computer simulation study. Neuroimage. Academic Press; 2016;137: 140–151. 10.1016/j.neuroimage.2016.05.032 27188218

[23] Bikson M, Datta A, Rahman A, Scaturro J. Electrode montages for tDCS and weak transcranial electrical stimulation: role of “return” electrode’s position and size. Clin Neurophysiol. NIH Public Access; 2010;121: 1976–8. 10.1016/j.clinph.2010.05.020 21035740 PMC2983105

[24] Wagner T, Fregni F, Fecteau S, Grodzinsky A, Zahn M, Pascual-Leone A. Transcranial direct current stimulation: A computer-based human model study. Neuroimage. 2007;35: 1113–1124. 10.1016/j.neuroimage.2007.01.027 17337213

[25] Nilsonne G, Tamm S, d’Onofrio P, Thuné HÅ, Schwarz J, Lavebratt C, et al A multimodal brain imaging dataset on sleep deprivation in young and old humans Inst för klinisk neurovetenskap / Dept of Clinical Neuroscience; 2016; Available: https://openarchive.ki.se/xmlui/handle/10616/45181

[26] Opitz A, Paulus W, Will S, Antunes A, Thielscher A. Determinants of the electric field during transcranial direct current stimulation. Neuroimage. 2015;109: 140–150. 10.1016/j.neuroimage.2015.01.033 25613437

[27] Windhoff M, Opitz A, Thielscher A. Electric field calculations in brain stimulation based on finite elements: An optimized processing pipeline for the generation and usage of accurate individual head models. Hum Brain Mapp. 2013;34: 923–935. 10.1002/hbm.21479 22109746 PMC6870291

[28] Rahman A, Lafon B, Bikson M. Multilevel computational models for predicting the cellular effects of noninvasive brain stimulation. Prog Brain Res. Elsevier; 2015;222: 25–40. 10.1016/bs.pbr.2015.09.003 26541375

[29] Elsner B, Kugler J, Pohl M, Mehrholz J. Transcranial direct current stimulation (tDCS) for improving aphasia in patients with aphasia after stroke Elsner B, editor. Cochrane Database Syst Rev. John Wiley & Sons, Ltd; 2015;25: CD009760 10.1002/14651858.CD009760.pub3 25929694

[30] Sandars M, Cloutman L, Woollams AM. Taking sides: An integrative review of the impact of laterality and polarity on efficacy of therapeutic transcranial direct current stimulation for anomia in chronic poststroke aphasia. Neural Plast. 2016;2016: 1–21. 10.1155/2016/8428256 26819777 PMC4706968

[31] Sebastian R, Tsapkini K, Tippett DC. Transcranial direct current stimulation in post stroke aphasia and primary progressive aphasia: Current knowledge and future clinical applications. NeuroRehabilitation. 2016;39: 141–152. 10.3233/NRE-161346 27314871 PMC4964590

[32] Galletta EE, Cancelli A, Cottone C, Simonelli I, Tecchio F, Bikson M, et al Use of computational modeling to inform tDCS electrode montages for the promotion of language recovery in post-stroke aphasia. Brain Stimul. 2015;8: 1108–1115. 10.1016/j.brs.2015.06.018 26198364

[33] Parazzini M, Fiocchi S, Liorni I, Ravazzani P. Effect of the interindividual variability on computational modeling of transcranial direct current stimulation Comput Intell Neurosci. Hindawi Publishing Corporation; 2015; 963293 10.1155/2015/963293 PMC4523656 26265912

[34] Pisoni A, Mattavelli G, Papagno C, Rosanova M, Casali AG, Romero Lauro LJ. Cognitive enhancement induced by anodal tDCS drives circuit-specific cortical plasticity. Cereb Cortex. 2018;28: 1132–1140. 10.1093/cercor/bhx021 28184424

[35] Turi Z, Mittner M, Paulus W, Antal A. Placebo intervention enhances reward learning in healthy individuals. Sci Rep. Nature Publishing Group; 2017;7: 41028 10.1038/srep41028 28112207 PMC5253628

